# Nanostructured Catalyst Layer Allowing Production of Ultralow Loading Electrodes for Polymer Electrolyte Membrane Fuel Cells with Superior Performance

**DOI:** 10.1021/acsaem.3c01987

**Published:** 2023-12-05

**Authors:** Colleen Jackson, Michalis Metaxas, Jack Dawson, Anthony R. Kucernak

**Affiliations:** Department of Chemistry, Imperial College London, White City, London W12 0BZ, United Kingdom

**Keywords:** Electrocatalysis, Catalysis, High utilization, electron transport, Nanoporous

## Abstract

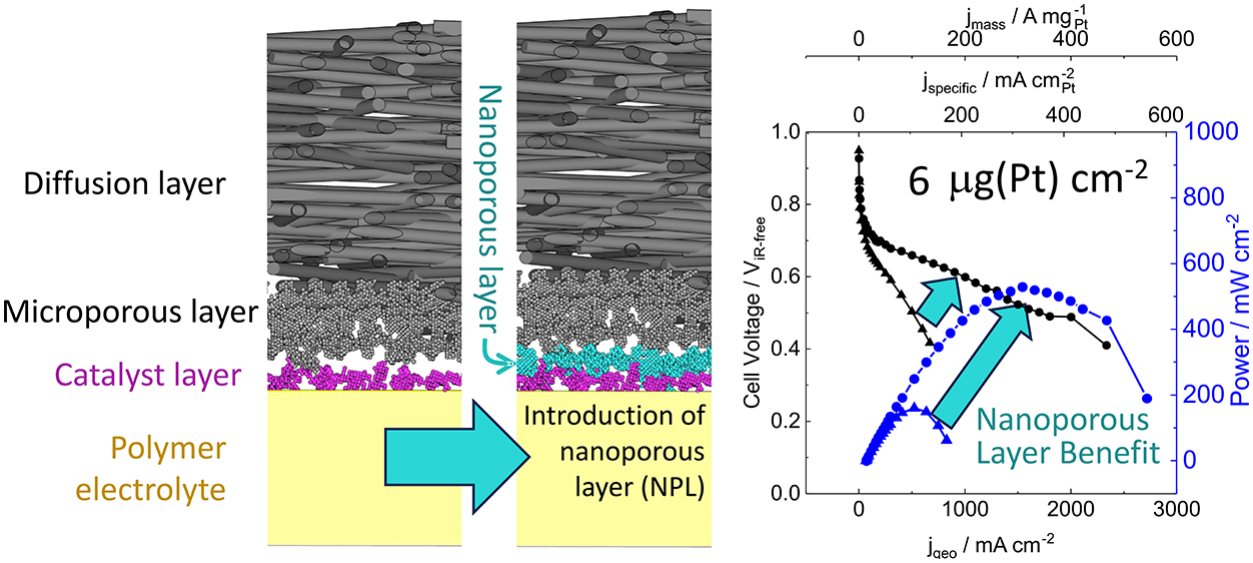

This study introduces a simple method to produce ultralow loading catalyst-coated membrane electrodes, with an integrated carbon “nanoporous layer”, for use in polymer electrolyte membrane fuel cells or other electrochemical devices. This approach allows fabrication of electrodes with loadings down to 5.2 μg_Pt_ cm^–2^ on the anode and cathode (total 10.4 μg_Pt_ cm^–2^, Pt_3_Zn/C catalyst) in a controlled, uniform, and reproducible manner. These layers achieve high utilization of the catalyst as measured through electrochemical surface area and mass specific activities. Electrodes composed of Pt/C, PtNi/C, Pt_3_Co/C, and Pt_3_Zn/C catalysts containing 5.2–7.1 μg_Pt_ cm^–2^ have been fabricated and tested. These electrodes showed an impressive performance of 111 ± 8 A mg_Pt_^–1^ at 0.65 V on Pt_3_Co/C with a power density of 31 ± 2 kW g_Pt,total_^–1^, about double that of the best previous literature electrodes under the same operating conditions. The performance appears apparently mass transport free and dominated by electrokinetics over a very wide potential range, and thus, these are ideal systems to study oxygen electrokinetics within the fuel cell environment. The improved performance is associated with reduced “contact resistance” and more specifically a reduction in the resistance to lateral current flow in the catalyst layer. Analytical expressions for the effect illuminate approaches to improve electrode design for electrochemical devices in which catalyst utilization is key.

## Introduction

In order to reduce the cost of the polymer electrolyte membrane fuel cell (PEMFC), considerable efforts have gone into understanding and improving the oxygen reduction reaction activities so that the amount of Pt in the catalyst layer can be reduced. A state of the art stack achieves 6.8 kW g_Pt,total_^–1^ (in 2016),^1^ where the Pt catalyst and application contributes to ∼21%–41% of the stack cost for 1000–500,000 systems/year (in 2015).^2^ The current Clean Hydrogen Partnership PEMFC targets aim to reduce the Pt content by >77% to >12.5 kW g_Pt,total_^–1^ by 2024 and further to >20 kW g_Pt,total_^–1^ by 2030,^3^ while increasing the mass activity to 15 A mg^–1^_Pt_ from 4.5 A mg^–1^_Pt_ at 0.66 V.^4^

Ultralow loading PEMFC research has shown to increase the catalyst utilization and increase the mass specific activities^5^ and power densities;^6−9^ however, a challenge arises in producing uniform catalyst layers with Pt loadings of <10 μg_Pt_ cm^–2^ with Pt-supported catalysts. Even if such loadings can be achieved, they require sophisticated approaches (e.g., ultrasonic spray deposition) which require a large excess of catalyst due to coating inefficiency. Thus, significant quantities of catalyst are required to perform tests under PEMFC conditions, limiting the ability to determine the performance of the new electrocatalysts. Additionally, the rotating disc electrode technique, typically used for kinetic studies and catalyst activity screening with low loadings (∼5–20 μg_Pt_ cm^–2^) of fuel cell catalysts, is limited to a small potential window of 0.85–0.95 V due to high mass transport limitations, and often performance is not translated to fuel cells.^10^ Therefore, an ultralow loading, high mass transport technique to probe kinetic activity of catalysts is desirable.

**Figure 1:**
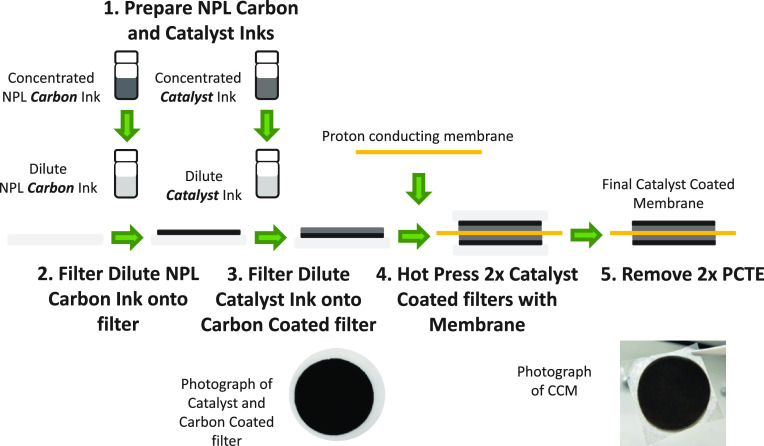
Schematic of catalyst-coated membrane preparation method. Schematic of the preparation procedure, including a photograph of the catalyst + carbon coated PCTE and the final catalyst-coated membrane.

**Figure 2:**
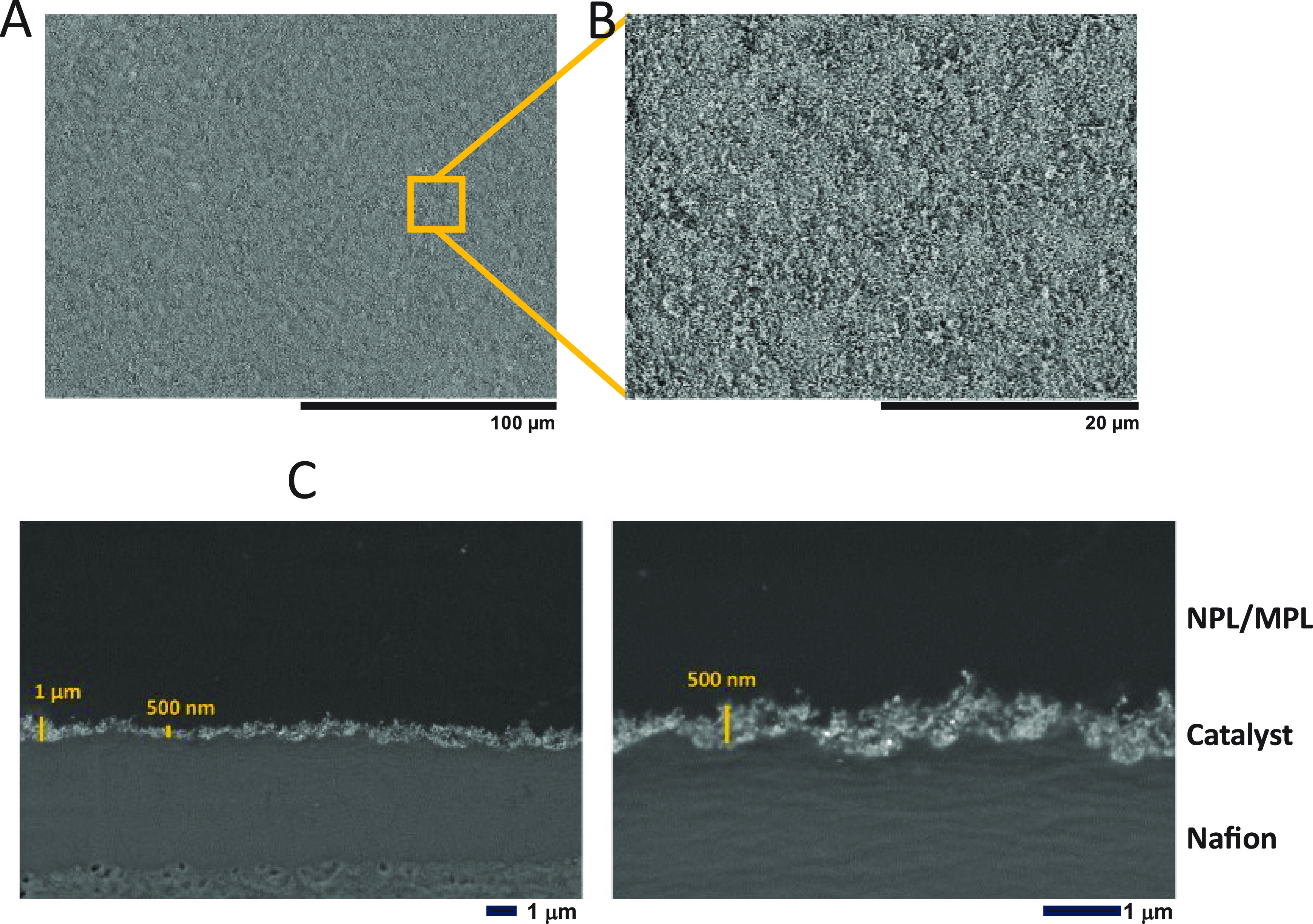
SEM images showing uniform coverage of the catalyst layers. (A, B) SEM overhead images of the catalyst and NPL coated membrane with two different magnifications. (C) Cross sectional view of 13.9 ± 0.3 μg_Pt_ cm^–2^ catalyst-coated membrane, after use in the fuel cell showing the catalyst layer with thickness between 0.5 and 1 μm. No boundary is visible between the NPL and the MPL.

This paper describes a new preparation method for ultralow loading catalyst layers (5.2–7.1 μg_Pt_ cm^–2^) with integrated carbon “nanoporous” layers (NPLs) which are produced by a filtration approach followed by decal transfer onto the proton conducting membrane. A schematic of the preparation method is shown in Figure 1 and is described in detail in the Methods section. An important benefit of this approach is that it leads to uniform deposition of catalyst even at ultralow loading. SEM images of the CCMs with integrated NPL show uniform coverage of the catalyst layer on the membrane in overhead images (Figure 2A, B; Figure S1). The amount of catalyst deposited on the electrode is controlled by the amount of catalyst in the filtrate solution (see Methods). The filtration process is **self-leveling** and produces a very uniform deposition across the entire area of the filter. Loss of catalyst through the filtration medium is mitigated by using track-etched filter membranes with uniform and small pore sizes (400 nm diameter) and having a prefiltered layer of carbon (which becomes the NPL) to act as a secondary filtration medium. The cross section of a CCM with 13.9 ± 0.3 μg_Pt_ cm^–2^ after fuel cell use shows uniform catalyst layer thickness in the range 0.5–1 μm (Figure 2C). Measurement of layers in CCMs before use give a value of total thickness of catalyst layer + NPL of 1.2 ± 0.2 μm (*n* = 10) (Figure S1C). X-ray fluorescence of CCMs with NPL layers measured loadings of 5.2–7.1 μg_Pt_ cm^–2^ on anode and cathode (10.4–14.2 μg_Pt_ cm^–2^ in total, see Methods section).

The approach allows efficient transfer of catalysts to enable the testing of low quantities of new catalysts. For instance, using a total of **5 mg** catalyst in making the inks, it is possible to produce around twelve 5 cm^2^ fuel cell CCMs. Furthermore, the low-loading nature of these catalysts allows electrochemical characterization, such as polarization curves of the catalysts under a wide range of conditions and extraction of important electrokinetic parameters. Such measurements are not possible with contemporary electrodes with 20 to 50 times the loading due to the confounding issues of water buildup and reactant mass transport.

## Results and Discussion

Production of the CCMs involves filtration processes that first deposit a carbon layer, acting as a nanoporous layer (NPL), and allow effective trapping of the subsequently filtered catalyst. The use of an ultraflat track-etched filter membrane contributes to uniform deposition of carbon and catalyst and allows production of low-loading electrodes across a wide area. The carbon NPL layer also allows for protection of the catalyst layer during the removal of the PCTE layers. Thus, any filtered material which remains attached to the PCTE is carbon, and the PCTE removal does not affect the catalyst loading (see Figure S2 for cartoon comparing catalyst deposition with and without NPL). Further, the in-plane electronic sheet resistance is decreased by ∼280× from 1.3822 ± 0.0005 MΩ square^–1^ without an NPL to 4.7985 ± 0.0002 kΩ square^–1^ with an NPL (see Methods). This large difference in resistance cannot be solely associated with the decreased thickness of the catalyst layer; for a homogeneous layer, a thickness reduction of a factor of ∼6× would be expected to increase the sheet resistance by the same factor as the sheet resistance is inversely proportional to the thickness. So, in this case, the resistivity of the layer must also vary with thickness. This is not such an unreasonable expectation as ultrathin layers may start approaching the percolation limit for electrical contact (see Figure S1 for SEM images of catalyst layers). For comparison, a typical catalyst layer with 200 μg_Pt_ cm^–2^ loading and a thickness of 7.0 ± 0.3 μm shows a sheet resistance of 0.9205 ± 0.0002 kΩ square^–1^, about 5 times lower than the catalyst layer with NPL (see Section S4).

### Effect of In-Plane Catalyst Layer Resistance on Current Collection

**Figure 3:**
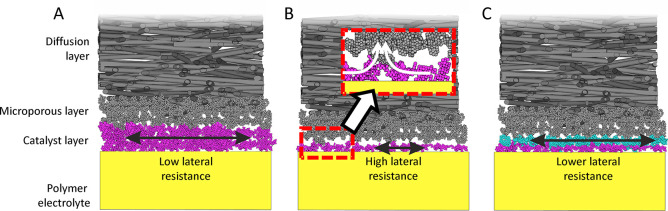
Cartoon comparing the case of an electrode with (A) thick and (B) thin catalyst layers and (C) case where a “nanoporous” layer is used to reduce lateral resistance.

In those cases where the catalyst layer is ultrathin (as seen in reduced catalyst loading electrodes), or the resistivity of the catalyst layer is high (due to the material choice in the layer), or the interface between the microporous layer (MPL) and catalyst layer is relatively rough (leading to areas where there is no electrical contact between the catalyst layer and the MPL), the in-plane flow of current may see an added electrical resistance. This phenomenon has been reported previously for ultralow loading PEM water electrolysis systems.^11,12^ Hence, decreasing the lateral sheet resistance of the catalyst layer is important for thin catalyst layers to avoid in-plane Ohmic loses, as the electronic (and ionic) current flows through the catalyst layer to make its way into the microporous layer (or membrane). As the catalyst layer becomes thinner, the sheet resistance^a^ increases, leading to large lateral Ohmic loses, as illustrated in the cartoon in Figure 3 and experimentally in the results presented below. Figure 3 shows a cartoon of the cross-section of a catalyst layer for a typical electrode, Figure 3A, in which lateral current flow to reach contact points between the catalyst layer and microporous layer is facile because of the relatively thick catalyst layer. In comparison, when the catalyst layer is thin, Figure 3B, a low density of interparticle contacts between catalyst particles contributes to a high lateral resistance and poor current collection. By including an extra “nanoporous layer” directly integrated with the catalyst layer (between the catalyst layer and the MPL), as in Figure 3C, lateral current collection is improved. The extra resistance seen by the current flowing between the catalyst layer and then into the microporous layer is dependent on the number of contact points between the two layers (a value which increases as the layers are made flatter) and the lateral resistance of the layers. The requirement of a lateral current flow occurring on relatively small distance scales of ∼10 μm in the catalyst layer leads to an extra electronic resistance, and this effect may be misinterpreted as a “pure” contact resistance. However, this effect is different from the “pure” contact resistance effect (which is independent of layer thickness) and becomes quite significant when catalyst layers become ultrathin or when contact points to the catalyst layer increase in separation due to a less smooth MPL or lower compression.

The areal extra resistance associated with lateral electrical current flow in the catalyst layer as a function of the sheet resistance of the layer (*R*_sheet_) is estimated by using eq 1 (see Section S5 for derivation^b^):
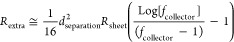
1where *R*_extra_ (Ω m^2^) is the extra areal resistance seen due to the lateral current flow and which is proportional to *R*_sheet_; *R*_sheet_ (Ω square^–1^) is the sheet resistance of the film; *d*_separation_ (m) is the distance between points of contact between the catalyst layer and the MPL, and *f*_collector_ is the fractional area of the contact through which the current flows to the MPL (0 < *f*_collector_ < 1). The bracketed term tends to zero as *f*_collector_ → 1 (i.e., as lateral current flow disappears) because the interfacial contact between MPL and catalyst layer is maximized. The term tends toward infinity as *f*_collector_ → 0, i.e., as local lateral current density close to the collection area becomes very large leading to a large lateral *iR* drop (see Section S4 for derivation). Determination of *f*_collector_ for real systems might be somewhat difficult and will be dependent on compression, but could be estimated using high resolution X-ray tomography of catalyst layers.^13^ As *d*_separation_ decreases, the number of contact points between MPL and catalyst layer increases quadratically, and so the extra areal specific resistance decreases in a quadratic manner. It might be possible to estimate *d*_separation_ from X-ray tomography or using the FIB/SEM reconstruction technique.^14^*R*_extra_ would appear as an extra area specific resistance in experimental results (e.g., see below where there is a quadrupling of that resistance) and explains common behavior seen in electrochemical devices such as fuel cells and electrolyzers where increased compression leads to a marked decrease in so-called “contact resistance”. This reduction in contact resistance can then be ascribed to (a) an increase in the number of contact points between the two layers (*d*_separation_ decreases as compression pressure increases) and (b) an increase in the contact area between the two layers (*f*_collector_ increases as the compression pressure increases). Both of these effects are nonlinear, and *R*_extra_ is expected to be quite nonlinear with compression effects. Model systems could be used to study these effects, e.g., systems in which contact to a catalyst layer is made through multiple microelectrodes of defined diameter and uniform separation in order to fix *d*_separation_ and *f*_collector_. This approach highlights the need for careful design of catalyst layers in which electrical conductance might be low, e.g., in the case of iridium oxide or other anode catalysts in water electrolyzers which show high resistivity, where there are moderate distances between the contact points to the catalyst layer (e.g., large pore sizes in the contacting current collector, or a rough current collector), or where the catalyst layer is ultrathin. This effect might also be important for ionic conduction in the catalyst layer too, although it is usually assumed that there is intimate contact between the membrane and ionomer, and so the need for **lateral** ionic current flow is small. As seen below, introduction of an added carbon layer on top of the catalyst layer can circumvent the problems of high lateral resistance and allow high utilization of catalysts, improving performance and leading to high mass activities.

### Fuel Cell Polarization Curves

**Figure 4:**
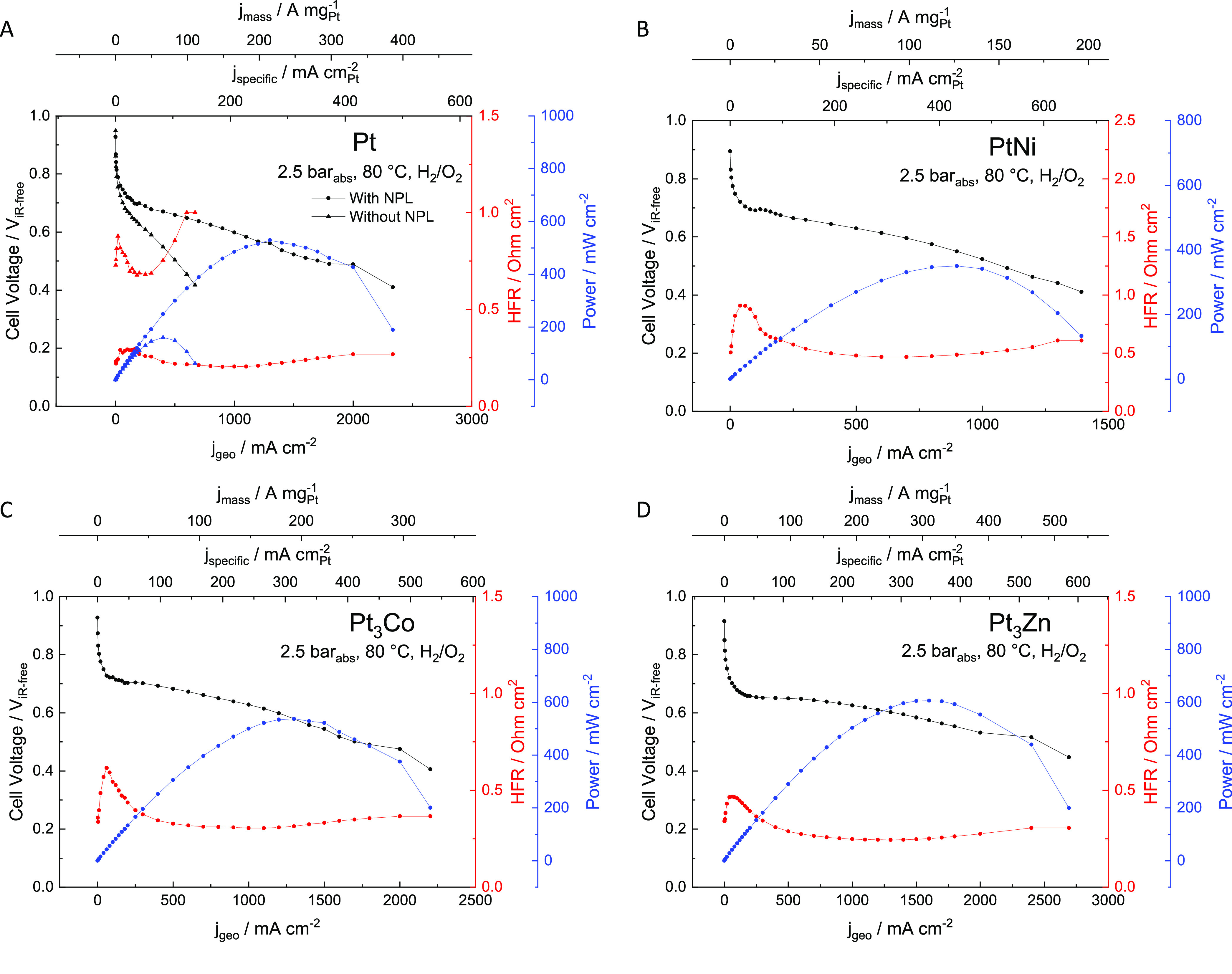
Polarization curves for the ultralow loading CCMs (5.2–7.1 μg_Pt_ cm^–2^ anode/cathode) under H_2_/O_2_. Polarization curves measured in H_2_/O_2_ at 2.5 bar_abs_ on the anode and cathode, at 80 °C with 100% and 75% RH on the anode and cathode, respectively. (A) Demonstrates the Pt CCM preparation with and without the NPL, where *j*_mass_ is based on Pt loading with a NPL. (B–D) NPL is included in preparation of PtNi, Pt_3_Co, and Pt_3_Zn CCMs.

The polarization curves, including geometric current (*j*_geo_), mass specific current (*j*_mass_), surface area specific current (*j*_specific_), high frequency resistance (HFR), and power density for Pt, PtNi, Pt_3_Co, and Pt_3_Zn catalysts are reported in Figure 4, with H_2_ (2.5 bar_abs_)/O_2_ (2.5 bar_abs_). The high frequency resistance of cells used to correct the cell voltage was around 250 mΩ cm^2^ for Pt, Pt_3_Co, and Pt_3_Zn electrodes with an NPL, associated with the use of a 50 μm thick Nafion 212 membrane. In all cases, there is a slight increase of the HFR at high current densities. It is unclear what is the reason for this divergence and is something that will be examined in a future paper including full electrochemical impedance spectroscopy results of these electrodes. The CCM with the PtNi catalyst (with NPL) showed a doubled HFR at 500 mΩ cm^2^ likely associated with nickel dissolution from the catalyst decreasing conductivity of the ionomer. The performance of an electrode with the same platinum loading but **without** an NPL is shown in Figure 4A, where the current densities are significantly smaller by 4-fold at 0.65 V, and the HFR is approximately 4-fold higher than the Pt electrode with an NPL, likely due to low in-plane conductivity as described above and some possible loss of Pt during preparation. Further, the electrochemical surface area of the catalyst layer **without** an NPL is 88% of that measured with an NPL, and the Pt loading was 66% lower since Pt was lost during decal transfer (see Section S1). The decrease in electrochemical surface area is then associated with lack of electrical contact to the Pt/C catalyst particles and by the loss of Pt during preparation if no NPL is used (i.e., not all Pt/C is transferred during CCM preparation when no NPL is used; see cartoon in Figure S2). If the change in HFR is associated with an increase in sheet resistance of the catalyst layer, eq 1 can be used to estimate the mean separation distance between contact points. Moreover, if it is assumed that the porosity of the layers is maintained at the interface, this suggests that *f*_collector_ must be quite low, of the order of 0.1 (i.e., a porosity of ≤0.9), and using the change in measured area specific resistance, *d*_separation_ is calculated to be about 20 μm, which is approximately twice the thickness of a normal catalyst layer, but more than 10 times greater than the thickness of the electrodes with an NPL, strongly supporting the idea that lateral current flow is important.

**Figure 5:**
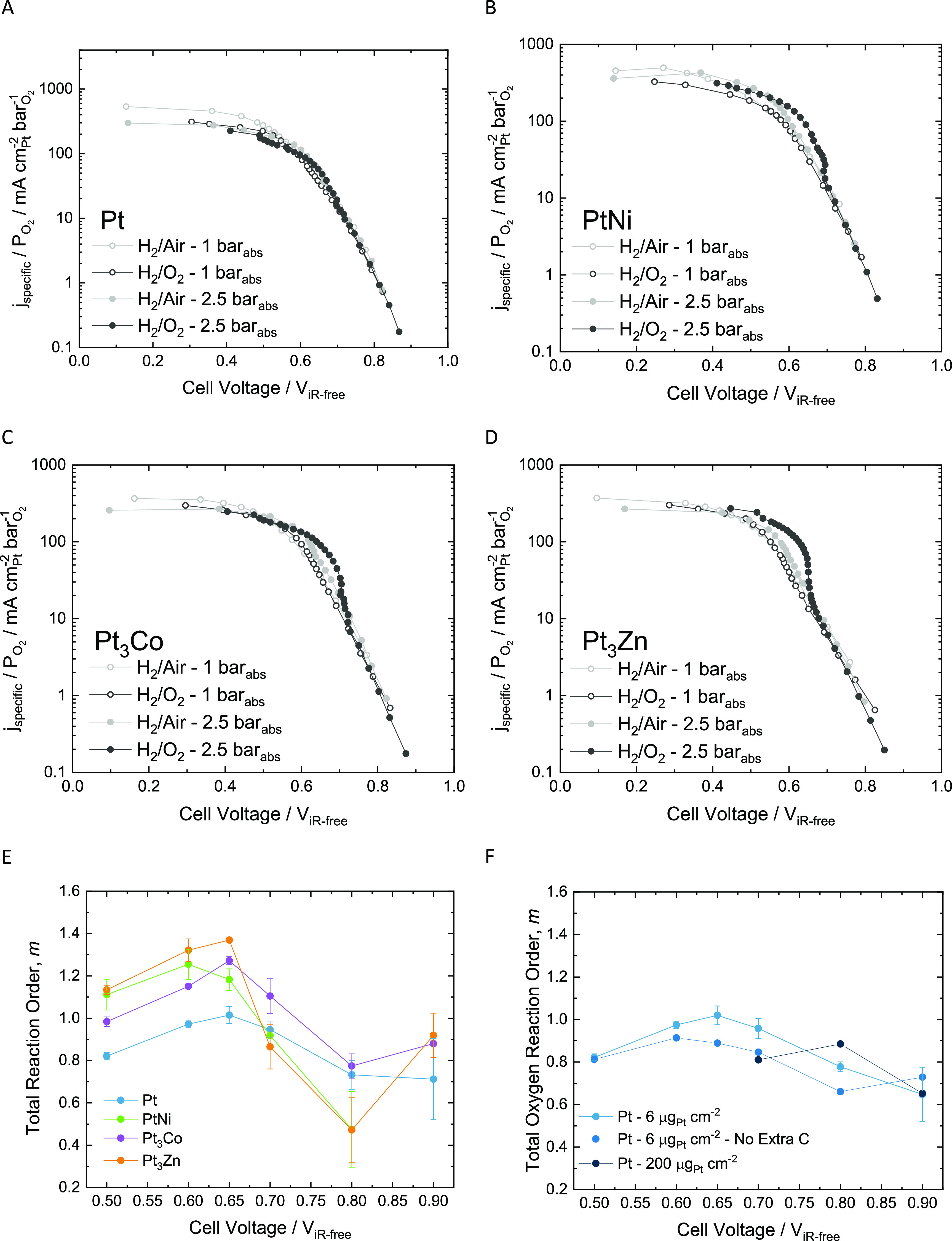
Normalized polarization curves for ultralow loading CCMs and total reaction orders. Oxygen partial pressure normalized polarization curves measured in H_2_/Air at 1 and 2.5 bar_abs_ and H_2_/O_2_ at 1 and 2.5 bar_abs_ on the anode and cathode, at 80 °C with 100% and 75% RH on the anode and cathode, respectively. Loading of 6 μg_Pt_ cm^–2^ for anode and cathode, with cathode composed of (A) Pt, (B) PtNi, (C) Pt_3_Co, and (D) Pt_3_Zn. (E) Total reaction orders (*m*) calculated at varying cell voltages for the four catalysts. (F) Total reaction orders (*m*) calculated at varying cell voltages for the low loading Pt, high cathode loading Pt (200 μg_Pt_ cm^–2^), and low loading Pt without the NPL. The polarization curves were corrected for water partial pressure and H_2_ crossover before determining the reaction order.

Polarization curves with varying oxygen partial pressures and total pressure are shown in Figure 5, in which the specific current density (i.e., current density normalized for the electrochemically active surface area) is divided by the oxygen partial pressure (corrected for water vapor) which varies by over 1 order of magnitude between the different experiments. In all cases, there is a transition to a limiting current at *j*_specific_/*P*_O2_ of ∼200 mA cm^–2^ bar_O2_^–1^. The similarity of these limiting currents argues against the limiting current being associated with water production as the absolute current density varies by over an order of magnitude. These polarization curves were used to determine the total oxygen reaction order (*m*), which is the dependence of current on the oxygen partial pressure at a constant cell voltage.
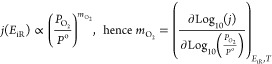
2

The oxygen reaction order is an important parameter in electrokinetic modeling of reactions as it contains useful kinetic information about the reaction slow step. The total oxygen reaction order was determined at different cell voltages for different catalysts and is shown in Figure 5E. Here, a range of total reaction orders from 0.4–1.4 were reported for the different catalysts; however, 0.9 V was less reliable due to hydrogen crossover and the low currents measured on the ultralow loading CCMs. The oxygen reaction order on Pt has previously been reported ex situ to be between 0.75–1 at 0.9 V vs RHE^15−17^ in acid electrolytes and 0.9–1 in situ between 0.65–0.75 V on an ultralow loaded inkjet printed electrodes of 26 μg_Pt_ cm^–2^.^18^ Therefore, these total reaction orders fall in line with previous literature values and show a potential dependence, generally reaching a peak at ∼0.65 V (at 1–1.4) for all the catalysts investigated. The high cathode loading (200 μg_Pt_ cm^–2^) and the Pt CCM without an NPL also show good agreement with the ultralow loading Pt CCMs (Figure 5F), although for the latter it is only possible to measure the reaction order over a more limited potential range due to the larger current density.

### Activities, Comparison to Literature and Reproducibility

**Figure 6:**
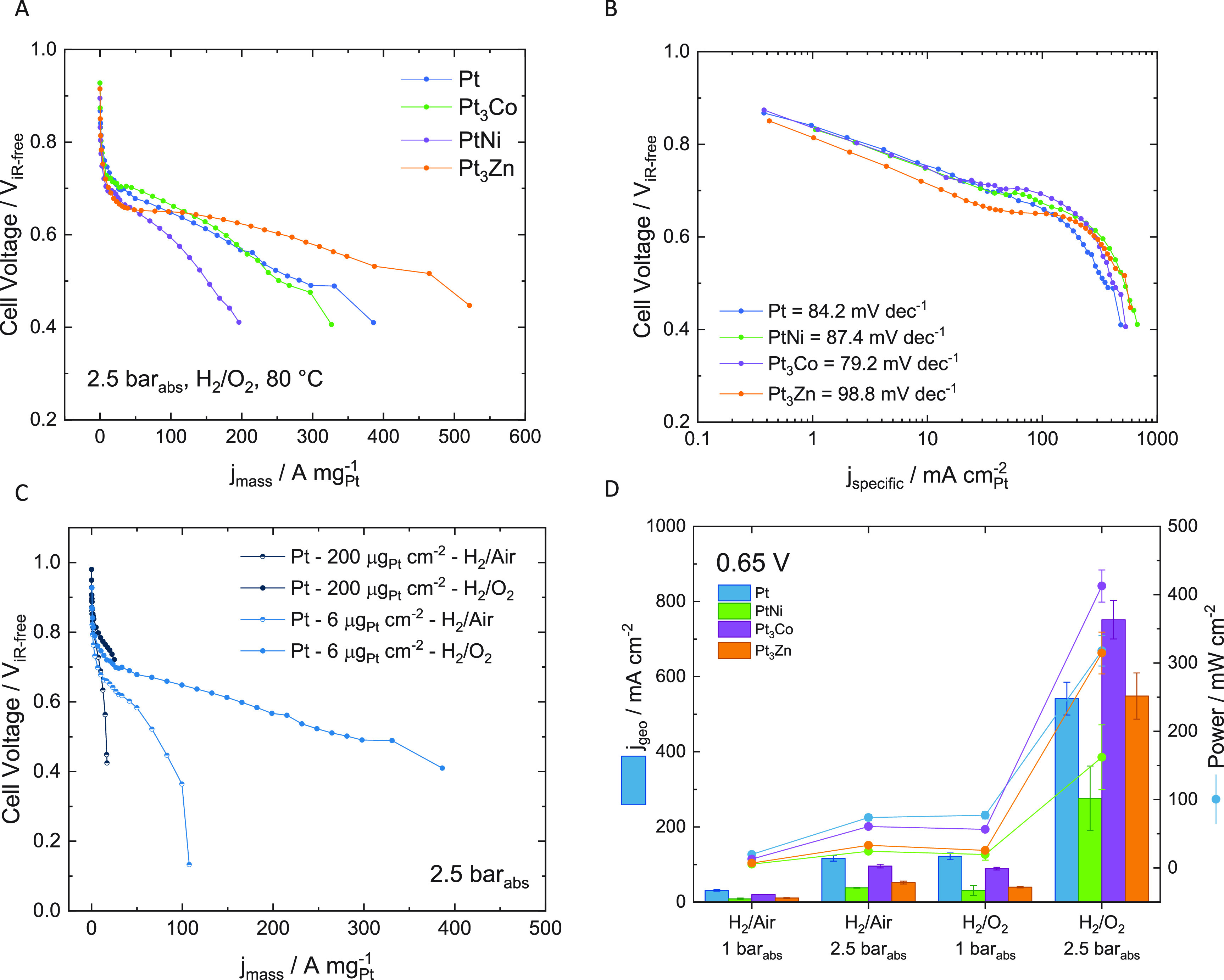
High performance polarization curves of ultralow loading CCMs. (A) Polarization curves measured in H_2_/O_2_ at 2.5 bar_abs_ on the anode and cathode, at 80 °C with 100% and 75% RH on the anode and cathode, respectively, where the *j*_mass_ is the cathode loading. (B) The surface area specific activity, calculated using ECSA of the cathode, of the polarization curves described in Figure 4A, where the Tafel slope values (linear fit for ∼0.3–50 mA cm^–2^_Pt_) for each catalyst is reported in the legend. (C) Polarization curves for the ultralow loading and high loading CCMs in H_2_/Air and H_2_/O_2_ at 2.5 bar_abs_ normalized for *j*_mass_. (D) Geometric current density (bar graph) and power density (line and symbol) at 0.65 V cell voltage, measured in H_2_/Air and H_2_/O_2_ at 1 and 2.5 bar_abs_.

The ultralow loading CCMs obtained a high current density of 0.8 A cm^–2^ at 0.65 V on the cathode coated with nominal loading of 6.7 μg_Pt_ cm^–2^ Pt_3_Co catalyst (Figure 6A and D), translating to a mass specific power density of 30.6 kW g_Pt,total_^–1^ or 0.033 g_Pt,total_ kW^–1^ where the total Pt nominal loading is 13.5 μg_Pt_ cm^–2^ (61.2 kW g_Pt_^–1^ or 0.016 g_Pt_ kW^–1^ for cathode only loading). Figure 6C compares the *j*_mass_ normalized polarization curves for the high loading and ultralow loading CCMs (all higher loading polarization curves are reported in the Figure S2); here, the ultralow loading CCMs outperform the high loading CCMs at cell voltages of <0.7 V, reaching 107 A mg_Pt_^–1^ and 386 A mg_Pt_^–1^ for air and O_2_ cathode gas, respectively. Cell voltages above 0.7 V are significantly affected by hydrogen crossover, which may explain the slightly lower *j*_mass_ on the ultralow loading CCMs compared to the higher loading electrode in the kinetic region.

The current densities and power outputs increased from polarization curves measured in H_2_/Air at 1 bar_abs_ to 2.5 bar_abs_, followed by further increases from H_2_/O_2_ at 1 bar_abs_ to 2.5 bar_abs_ (Figure 6D). Surprisingly, the Pt catalyst outperformed the Pt alloy catalysts using cathode gases of air at 1 bar_abs_ and 2.5 bar_abs_, as well as at low overpotentials under O_2_ at 1 bar_abs_. Pt_3_Co showed the highest performance with H_2_/O_2_ (2.5 bar_abs_) at 0.65 V, but at high overpotentials (cell voltages of <0.6 V) the Pt_3_Zn catalyst obtained higher current densities.

The Tafel slopes of all four CCMs follow a linear trend (Figure 6B) over almost **3** orders of magnitude of current, from 0.3–100 mA cm^–2^_Pt_, measuring low value of between 79–99 mV dec^–1^. Here, a linear slope across low current densities (LCD) to high current densities (HCD) to 50 mA cm^–2^_Pt_ at ∼0.65–0.72 V was observed. Previous studies, typically measured on RDEs, have shown Tafel slopes of 60 mV dec^–1^ at LCD, transitioning to 120 mV dec^–1^ at HCD.^19,20^ Previous work on inkjet-printed ultralow loading electrodes (26 μ_Pt_ cm^–2^) show a single linear Tafel slope of higher values (77–156 mV dec^–1^) over only 1 order of magnitude of current.^18^ However, in this work, no transition was observed, as gradients of 79–99 mV dec^–1^ were maintained for all four catalysts over almost 3 orders of magnitude of current, with the slopes being similar in value to the ultralow inkjet-printed electrodes, with a singular Tafel slope, but these Tafel slopes are lower than previous work on ultralow loading electrodes.

**Figure 7:**
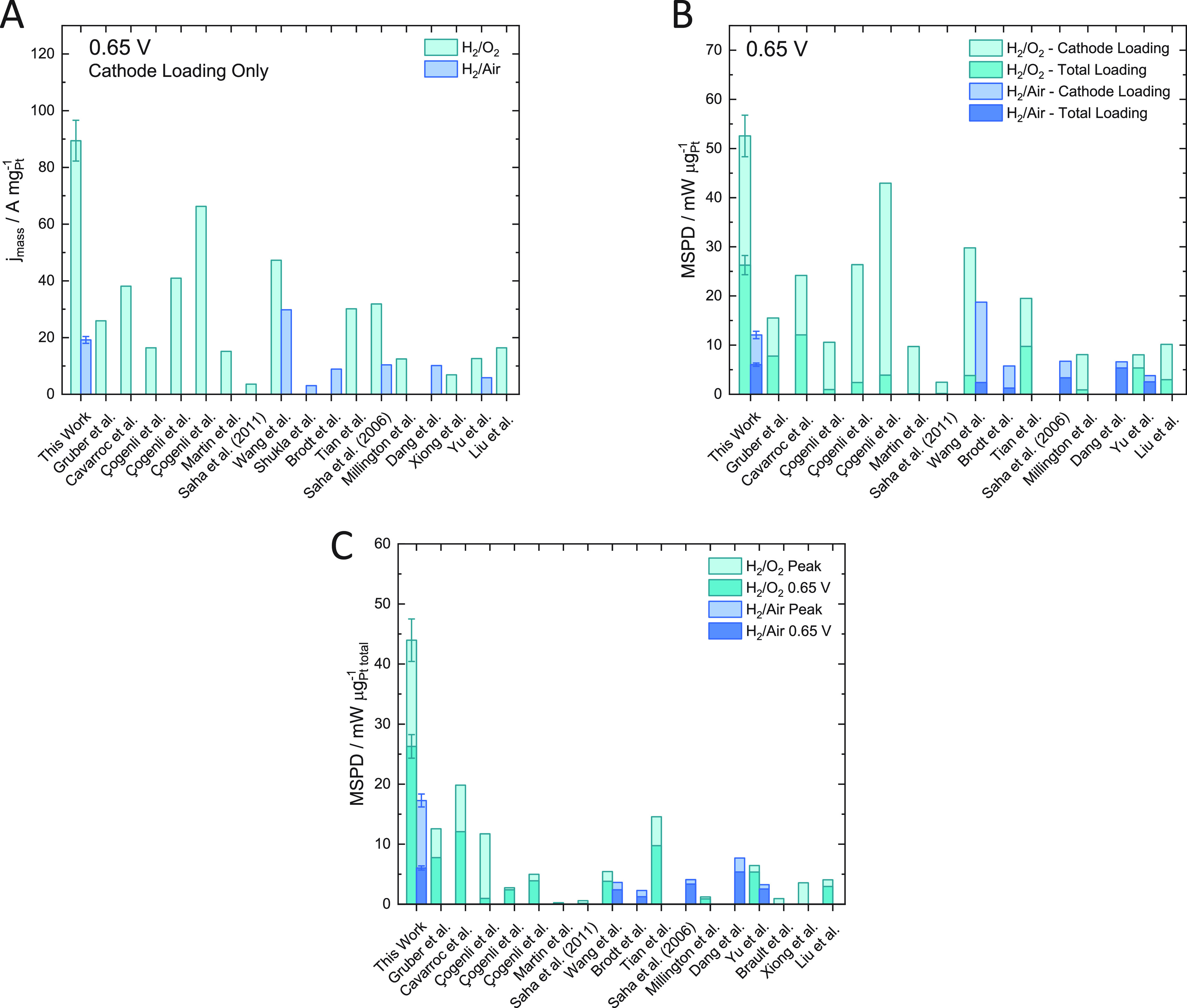
Comparison to literature for Pt catalysts. This work (H_2_/O_2_ and H_2_/Air at 2.5 bar_abs_) compared to literature values, Gruber et al.,^24^ Cavarroc et al.,^6^ Çögenli et al.,^21^ Martin et al.,^22^ Saha et al.,^25^ Wang et al.,^23^ Shukla et al.,^18^ Brodt et al.,^26^ Tian et al.,^8^ Saha et al.,^27^ Millington et al.,^28^ Dang et al.,^29^ Xiong et al.,^30^ Yu et al.,^31^ Brault et al.^32^ and Liu et al.^9^ (A) Mass specific current density at 0.65 V normalized by cathode Pt loading. (B) MSPD at 0.65 V with H_2_/O_2_ and H_2_/Air at 2.5 bar_abs_ normalized by cathode and total Pt loading. (C) Peak MSPD and MSPD at 0.65 V with H_2_/O_2_ and H_2_/Air at 2.5 bar_abs_ normalized by total Pt loading.

A literature comparison between mass specific current density at 0.65 V and peak mass specific power density (MSPD) and MSPD at 0.65 V for Pt catalysts (not including alloys) is given in Figure 7. In Figure 7A and B, the mass specific current density at 0.65 V and MSPD at 0.65 V are normalized by the nominal cathodic loading. Cathodic loading is used to normalize the activities and power densities since some studies have unnecessarily high anodic loadings, for instance Çogeni et al.^21^ use an anode loading which is 10× higher the cathode loading, and Martin et al.^22^ use an anode loading 83× higher than the cathode loading. Since the hydrogen oxidation reaction on the anode is a facile reaction, particularly in comparison to oxygen reduction on the cathode, significantly larger Pt anodic loadings are unnecessary. Therefore, in order to make a fairer comparison between literature values, both cathodic and total Pt loading normalization is shown in Figure 7B and C, where the total Pt loading normalization is reported to evaluate performance against the Clean Hydrogen Partnership (formerly the FCH JU) targets. In our study, we have determined total platinum loading by XRF and performed measurements on multiple electrodes to obtain a quantitative measure of reproducibility. Most literature results do not accurately quantify the amount of platinum in the catalyst layers, which is important for low loading systems, as small deviations in loading can make large differences in activity. Even fewer studies report the reproducibility of results, with most studies only providing one (presumably the best) result. In Section S7, we provide a table with a breakdown of low-loading production approaches, measurement approaches (direct, indirect or “dead reckoning”), and whether repeats were performed for the results presented in Figure 7.

The peak MSPD and MSPD at 0.65 V measured in this work are consistently higher than previously reported ultralow loading CCMs using H_2_/O_2_ feed gases, using both Pt cathode loading and Pt total loading normalization. With H_2_/Air feed gases, Wang et al.^23^ obtains slightly higher mass specific current densities and MSPD at 0.65 V when normalized by the cathode loading; however, Wang et al.^23^ used 150 μg_Pt_ cm^–2^ on the anode (22 μg_Pt_ cm^–2^ on the cathode), significantly lowering the performance when normalizing for total Pt loading. From the total Pt loadings normalized values, the CCMs produced in this work achieve more than double the peak MSPD (under both air and oxygen cathode gases) and MSPD at 0.65 V (under oxygen cathode gas) than previously reported in the literature (Figure 7C).

Moreover, this work obtains higher MSPD at 0.65 V compared to the commonly used high loading CCMs with 300 μg_Pt,total_ cm^–2^ of ∼3.5 kW g^–1^_Pt_ (or 0.28 g_Pt,total_ kW^–1^ for H_2_/Air at 2.5 bar_abs_). Additionally, the mass specific current densities measured on the ultralow loading Pt CCMs in H_2_/Air at 2.5 bar_abs_ of 5.1 kW g^–1^_Pt,total_ at 0.66 V are an improvement over the 2017 state of the art (2.5 kW g^–1^_Pt,total_) and approaching the Clean Hydrogen Partnership for 2024 of 12.5 kW g^–1^_Pt,total_ (or 0.08 g_Pt,total_ kW^–1^)^4^. It is reassuring to see that effective deposition of the catalyst can allow high performance without the requirement of more active catalysts.

The mass specific activities (also shown in Table 1 for all catalysts) at 0.65 V report high activities; specifically, Pt under H_2_/O_2_ (1 bar_abs_) of 20.1 A mg_Pt_^–1^ was similar to those previously measured on the floating electrode of ∼25 A mg_Pt_^–1^. The floating electrode technique probes the kinetic activity of catalysts, ex situ, under high mass transport conditions, thus illustrating our ultralow loading CCMs of ∼6 μg_Pt_ cm^–2^ were likely probing the kinetic performance of the catalysts, free of mass transport limitations, and issues of water buildup. Once more, the highest performance was measured on the Pt_3_Co under H_2_/O_2_ (2.5 bar_abs_) of 111 A mg_Pt_^–1^ at 0.65 V. It is interesting to note that the performance of the catalysts under pure oxygen (1 bar_abs_) is close to the performance under air at 2.5 bar_abs_. This effect may be associated with the similarity of oxygen partial pressure at these two operating conditions, especially if it is assumed that at 0.65 V the vapor in the catalyst is saturated (i.e., 100% RH rather than 75% RH of the cathode feed). This is further discussed in Section S8.

**Table 1:** Mass Activities at 0.65 V Cell Voltage^a^

Catalyst	H_2_/Air 1 bar_abs_ *j*_mass_ @ 0.65 V/A mg_Pt_^–1^	H_2_/Air 2.5 bar_abs_ *j*_mass_ @ 0.65 V/A mg_Pt_^–1^	H_2_/O_2_ 1 bar_abs_ *j*_mass_ @ 0.65 V/A mg_Pt_^–1^	H_2_/O_2_ 2.5 bar_abs_ *j*_mass_ @ 0.65 V/A mg_Pt_^–1^
Pt	5.2 ± 0.3	19.2 ± 1.2	20.1 ± 1.5	89.5 ± 7.2
PtNi	1.2 ± 0.3	5.4 ± 0.1	4.3 ± 1.9	38.8 ± 12.1
Pt_3_Co	3.0 ± 0.1	14.2 ± 0.7	13.2 ± 0.5	111.5 ± 7.6
Pt_3_Zn	2.1 ± 0.2	10.0 ± 0.7	7.7 ± 0.4	106.2 ± 11.9

a Mass activities under H_2_/Air and H_2_/O_2_ under 1 and 2.5 bar_abs_ pressure (on both the anode and cathode) are reported. Cell temperature was 80 °C with 100% and 75% RH on the anode and cathode, respectively.

## Conclusion

This work reports high performance ultralow loading CCMs of 5.2–7.1 μg_Pt_ cm^–2^, with an integrated “nanoporous” layer, for PEMFCs which achieve peak MSPDs and MSPD at 0.65 V (under oxygen) which are more than double those previously reported in literature. The use of the nanoporous layer is important in mitigating effects associated with lateral current flow in these ultrathin layers and also important in proton transport, leading to a 4-fold reduction in the area-specific resistance, which leads to a 4-fold improvement in current density at 0.65 V. The effect has been quantified and assessed though an analytical expression. The approach developed rationalizes the effects seen in real electrochemical systems in which increased compression reduces the observed areal specific resistance. A range of Pt catalysts (Pt/C, PtNi/C, Pt_3_Co/C, Pt_3_Zn/C) were investigated, achieving 38–111 A mg_Pt_^–1^ at 0.65 V under H_2_/O_2_ 2.5 bar_abs_, with ECSAs of 38.5–86.8 m^2^ g_Pt_^–1^ and good reproducibility. The polarization curves probe the kinetic performance of the catalysts under high mass transport conditions, showing linear Tafel slopes of 79–99 mV dec^–1^ over 3 orders of magnitude. The absence of a transition in Tafel slope as sometimes seen by others, especially in three-electrode cell measurements, might be associated with the paucity of data in our plots at high potentials or the presence of a small amount of hydrogen crossing over the membrane (although a thicker Nafion membrane was chosen to try and mitigate this effect). The measured MSPD at 0.65 V in H_2_/Air at 2.5 bar_abs_ was double that of the commonly used high loading CCMs (300 μg_Pt,total_ cm^–2^).

This CCM preparation technique did not require sophisticated equipment, such as a spray coater or inkjet printer, had high utilization of the catalyst, and may be used for a range of catalysts or devices. Moreover, a minimal amount of catalyst was used in the production of the CCMs and allows for layer design and thrifting on CCMs for electrochemical devices to be further investigated. A key component of the ultralow loading CCMs is an integrated carbon NPL which improves the uniformity of catalyst deposition and transfer onto the membrane, as well as the in-plane electronic conductivity of the catalyst layer.

It is intriguing to consider whether lateral ***ion*** flow (e.g., protons in our case) might also be an important factor in ultrathin catalysts layer especially as the bulk resistivity of ion flow in common ionomers is about 4 orders of magnitude **higher** than electronic transport in the materials used in these electrochemical devices.

Future work in this area should be to better understand the effects of catalyst loading on the anode and cathode as it is well established that due to the much higher exchange current density of the hydrogen reaction, loading on the anode can be much less than on the cathode. Furthermore, other effects such as the NPL layer thickness, ionomer to carbon ratio, and lamination conditions (temperature/pressure) may be modified to further improve the performance of these layers. Lower loading electrodes are liable to be more sensitive to catalyst loss through dissolution and poisoning effects and might be a useful system to study such affects.

## Methods

### Catalysts

The electrocatalysts used were as-received: Pt/C (50 wt % Pt on high surface area carbon) TKK TEC10E50E, 40 wt % PtNi/C (Pt:Ni ratio of 1:1, Fuel Cell Store), 40 wt % Pt_3_Co/C (Pt:Co ratio of 3:1, Premetek Co.), and 20 wt % Pt_3_Zn/C (Pt:Zn ratio of 3:1, Premetek Co.).

### Catalyst-Coated Membrane Preparation

Ultrathin electrode specifications are nominal 6 μg_Pt_ cm^–2^/6 μg_Pt_ cm^–2^ anode/cathode Pt loading (precise value determined by XRF) with an integrated NPL with a total catalyst-coated membrane (CCM) surface area of 5 cm^2^, using an ionomer:carbon ratio of 0.8:1 D2020 (Chemours), Nafion 212 membranes, and SGL 22 BB (215 μm, 5 wt % PTFE) GDL’s on the anode and cathode.

The catalyst layer was prepared via a modified floating electrode preparation method.^10^ Anode and cathode catalyst layers with integrated NPL were prepared using an NPL carbon ink and anode/cathode catalyst inks. For the NPL carbon ink, a concentrated stock ink was prepared, and from this, a dilute ink was used in the filtration process. The stock NPL carbon ink was prepared using 5 mg of Vulcan XC72R, (where the Vulcan XC72R was precleaned in aqua regia overnight and thoroughly washed with deionized water) in 6 mL of 3:1 v/v isopropanol:water; this was sonicated for 30 min before 5 wt % Nafion (Sigma-Aldrich) was added, and the concentrated ink was sonicated for a further 45 min. This produced an ink with a 0.8:1 ratio of carbon:Nafion ratio. Then, 633 μL of the concentrated carbon ink (corresponding to approximately 520 μg of carbon) was diluted with 15 mL of 3:1 v/v isopropanol:water, and this dilute ink was then vacuum filtered onto the 12 μm thick ultraflat 4.7 cm diameter polycarbonate track etched (PCTE) filtration membrane (Sterlitech, PCTF0447100, a pore diameter of 400 nm, a porosity of 0.125, pore tortuosity of 1, and a pore density of 10^8^ pores cm^–2^). The vacuum filtration was done in a laminar flow hood using HEPA filtered air to reduce the risk of contamination during the preparation of the electrodes. This produced an NPL layer comprising 30 μg_Carbon_ cm^–2^. A concentrated catalyst (Pt/C, PtCo/C, PtZn/C etc.) ink was prepared, using 5 mg of catalyst in 6 mL of 3:1 v/v isopropanol:water. This was sonicated for 30 min before 5 wt % Nafion (Sigma-Aldrich) was added (0.8:1 ratio of carbon:Nafion), and the concentrated ink was sonicated for a further 45 min. Then, 227 μL of the concentrated stock catalyst ink (corresponding to approximately 87 μg_Pt_ of catalyst) was diluted with 15 mL v/v of 3:1 isopropanol:water, and this dilute ink was then vacuum filtered onto the carbon NPL coated PCTE membrane. This was then repeated to form two catalyst layers (anode and cathode), which were subsequently hot pressed onto the Nafion 212 membrane at 140 °C for 20 min at 10 bar. The two PCTE filtration membranes were then peeled off to leave the CCM with integral NPL.

### Measurement of Catalyst Loading

X-ray fluorescence (Fischerscope X-ray XDV) was used to confirm the total Pt loading (anode + cathode), from four to nine points taken from one CCM. The Pt loadings for Pt/C with a NPL, Pt/C without a NPL, PtNi/C, Pt_3_Co/C, and Pt_3_Zn/C were measured to be 12.10 ± 1.20, 8.01 ± 0.67, 14.21 ± 0.41, 13.48 ± 0.36, 10.33 ± 0.32 μg_Pt_ cm^–2^, respectively, for the total loading, which is assumed to be 6.05 ± 0.6, 4.00 ± 0.33, 7.11 ± 0.21, 6.74 ± 0.18, and 5.16 ± 0.16 μg_Pt_ cm^–2^, respectively, for the anode/cathode loading.

### SEM Cross Section Analysis

For scanning electron microscopy (SEM) imaging of the electrode cross section, the MEA was embedded in epoxy resin and then mechanically polished to a mirror-like surface. The observations were performed with a ZEIS-LEO 1530 field emission gun microscope at 5 kV acceleration voltage and using the backscattered electron detector.

### Electrochemical Characterization

Serpentine flow fields were used with a 5 cm^2^ active area (Scribner Associates). The membrane electrode assemblies (MEAs) were compressed by 20% using gaskets. MEAs were humidified at 80/80/73 °C (cell temperature/anode/cathode) for 3 h with H_2_ (BIP Plus, Air Products) and N_2_ (BIP Plus, Air Products) flow to the anode and cathode, respectively, each with flow rates of 0.17 mL min^–1^. Break-in is performed by holding the cell voltage at 0.55 V for 3 h with a stoichiometry of 4 for air at the cathode and 3 for H_2_ at the anode. Polarization curves were obtained using the same stoichiometries, holding at each point for 2 min. High frequency resistance (HFR) at 1000 Hz was used to correct for iR.

Electrochemically active surface area (ECSA) was measured using CO stripping voltammetry at room temperature. The potential was held at 0.2 V vs RHE for 5 min in 1000 ppm of CO/N_2_ (BOC) and then 5 min purging with N_2_ (BIP Plus, Air Products), and cyclic voltammetry was measured with a potential range of 0.08–1.2 V with a scan rate of 50 mV.s^–1^.

### Electronic Conductivity

In-plane electronic resistance was determined by measuring hot-pressed catalyst layers using a 4-point probe resistance measurement using a Keithley 3706A System Switch/Multimeter operating in “Dry Circuit Resistance” mode. This resistance measurement mode keeps the applied potential difference to less than 27 mV to avoid the possibility of inducing electrolytic processes which would otherwise impair the measurement of the electronic resistance.

## Data Availability

The data used in the production of this paper is available for download at the following DOI: 10.5281/zenodo.10256698.

## Supporting Information Available

SEM images without nanoporous layer, normal loading CCM performance curves, and derivation of electrical collection in the presence of voids (PDF)

## Footnotes

a Sheet resistance is an easily measured parameter of homogeneous layers and is equal to the resistivity of the film multiplied by its thickness.

b This analysis does not consider the effect that the lateral potential drop will have on perturbing the local rate of reaction which might be significant if the potential drop is large.
