# Supplementary material for: Nanostructured Catalyst Layer Allowing Production of Ultralow Loading Electrodes for Polymer Electrolyte Membrane Fuel Cells with Superior Performance
Source: ACS Appl Energy Mater. 2023 Dec 5;6(24):12296–306. doi: 10.1021/acsaem.3c01987 (PMC10751738; doi:10.1021/acsaem.3c01987)
Supplement: Supplementary file 1 — ae3c01987_si_001.pdf [file ae3c01987_si_001.pdf]

Supporting Information

# Nanostructured Catalyst Layer Allowing Production of Ultra-Low Loading Electrodes for PEMFCs with Superior Performance

Colleen Jackson, Michalis Metaxas, Jack Dawson, and Anthony R. Kucernak*

*Department of Chemistry, Imperial College London, White City, London, UK, W12 0BZ*

**a.kucernak@imperial.ac.uk*

## S1. Electrochemically Active Surface Area (ECSA)

ECSAs for Pt of 79.9 ± 0.3 $m^2\ g_{Pt}^{-1}$ determined using CO adsorption and stripping are reported in Table S1, this is slightly below with benchmark values for this catalyst reported on the rotating disc electrode by Shinozaki *et al.* of 98 ± 6 $m^2\ g_{Pt}^{-1}$ [12] and 99 ± 5 $m^2\ g_{Pt}^{-1}$ [13]. The Pt alloys have ECSAs which range 38 – 87 $m^2\ g_{Pt}^{-1}$, all ECSAs have standard deviations of 2 – 13 % depending on the catalyst.

***Table S1: Electrochemically Active Surface Areas (ECSAs).*** *ECSAs of the catalysts, measured by CO Stripping Voltammetry using two different CCMs to determine error margins.*

| Catalyst | ECSA / $m^2\ g_{Pt}^{-1}$ |
|---|---|
| Pt | 79.9 ± 0.3 |
| PtNi | 38.6 ± 5.0 |
| $Pt_3Co$ | 58.7 ± 2.7 |
| $Pt_3Zn$ | 86.8 ± 2.4 |

## S1. SEM Imaging

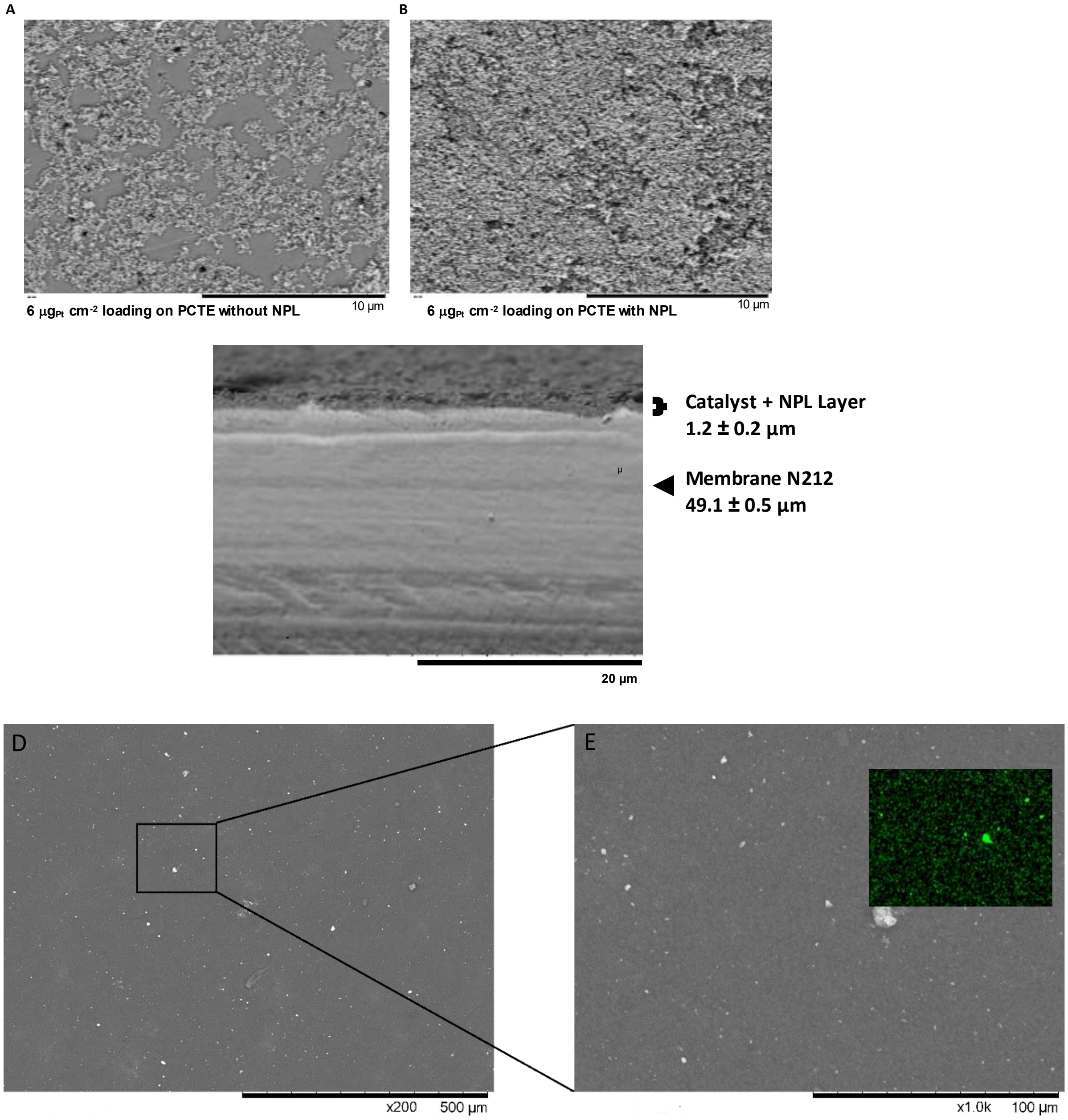


***Figure S1: SEM Images of low loading Catalyst Layers.*** *A) Top view of 6 μg$_{Pt}$ cm$^{-2}$ catalyst without a NPL filtered on PCTE and hot pressed, with 5 nm of Au sputtered to aid in conductivity for imaging. B) Top view of 6 μg$_{Pt}$ cm$^{-2}$ catalyst with a NPL filtered on PCTE and hot pressed, with 5 nm of Au sputtered to aid in conductivity for imaging. C) Cross section of 6 μg$_{Pt}$ cm$^{-2}$ catalyst with NPL layer hot pressed onto Nafion 212 electrolyte. SEM images of a 3 μg$_{Pt}$ cm$^{-2}$ electrode showing uniform coverage at (d) x200 and (e) x1000 magnification. (e) Inset – Pt EDX signal for x1000 image*

Figure S1a shows clear areas where the catalyst layer is not homogenous on the PCTE, leading to an extra resistance due to low in-plane conductivity of the catalyst layer. By including a NPL (Figure S1b), the layer is more uniform and there is better electrical contact.

## S2. Incorporation of NPL leads to no catalyst loss

The effect of the NPL is to allow more complete transfer of catalyst from the filter to the membrane as some material always remains adhered to the filter. When an NPL is used the adherent material is carbon whereas if one is not used the adherent material is catalysts leading to a loss of catalyst in the catalyst layer.

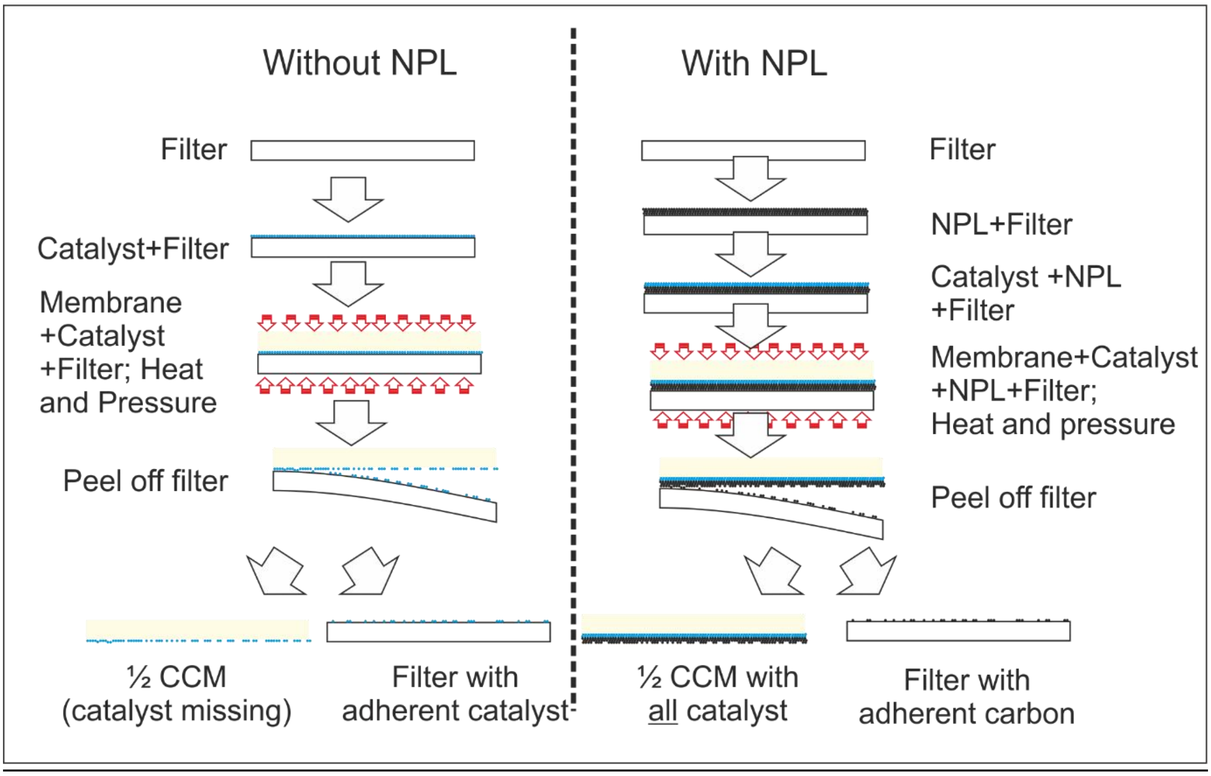


***Figure S2: Cartoon showing loss of catalyst from catalyst layer in absence of NPL layer.*** *Without an NPL (LHS) some catalyst remains on the filter whereas if an NPL is used, all the catalyst is transferred (RHS).*

## S3. Normal Loading Polarisation Curves

The higher loading CCMs are 100/200 μg$_{Pt}$ cm$^{-2}$ anode/cathode loading of Pt/C (TEC10E50E), the polarisation curves are shown in Figure S3.

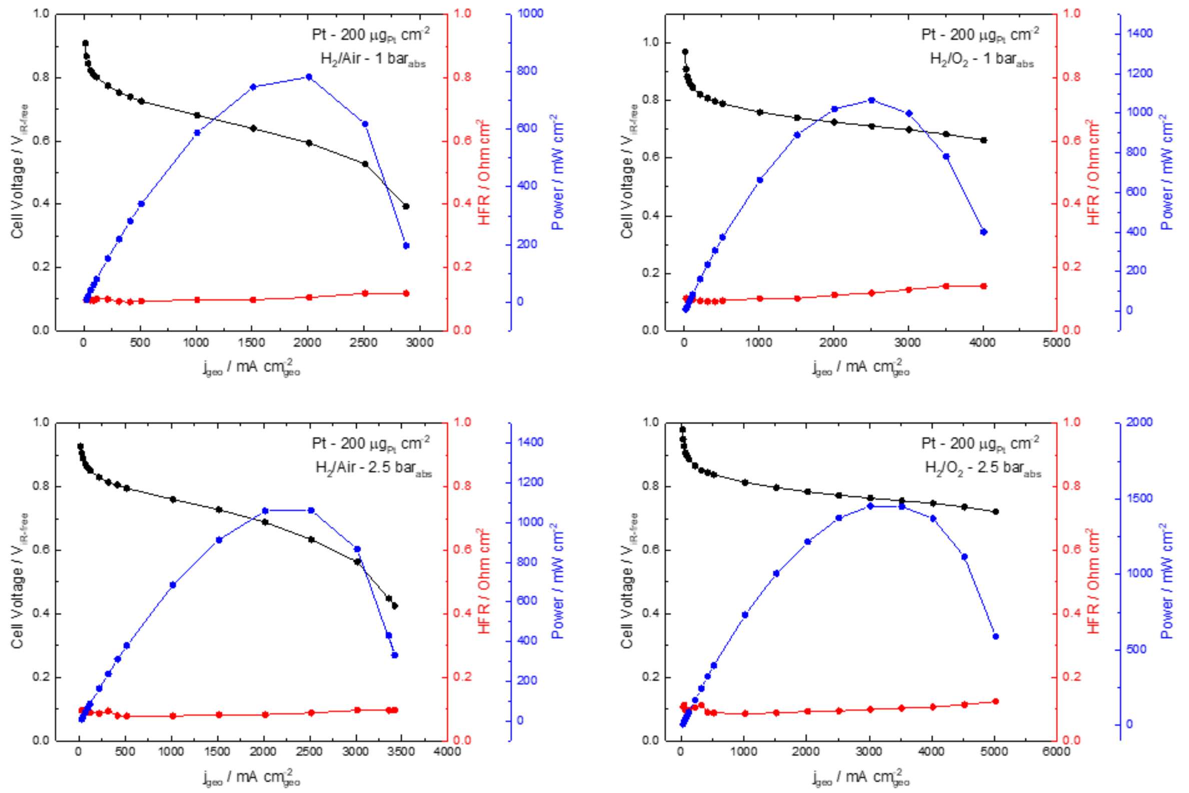


***Figure S3:*** *Polarisation curves of the high loading CCM. Measured in $H_2$/Air and $H_2/O_2$ at 1 and 2.5 bar$_{abs}$ on the anode and cathode, at 80 °C with 100% and 75% RH on the anode and cathode, respectively.*

## S4. Electrical conductivity of normal loading electrodes

In order to measure the electrical resistivity of a catalyst layer it is important to not have it in contact with electrolyte to gas diffusion media. Hence we deposited the catalyst layer on a ptfe film and exposed it to the same conditions as a catalyst layer would see when undergoing lamination to the membrane. A catalyst layer containing are 200 μg$_{Pt}$ cm$^{-2}$ of Pt/C (TEC10E50E) as used above was deposited onto a ptfe blank and pressed at the same temperature, pressure and time as that used to produce the electrodes used in Figure S3. This catalyst layer was then used to measure the electrical resistivity of the catalyst layer using 4-probe resistance measurement. An SEM image of the catalyst layer on the ptfe film is shown in Figure S4. Measurement of the thickness of the catalyst layer was performed at 17 positions and provided a thickness of 7.0±0.3 μm (n=17).

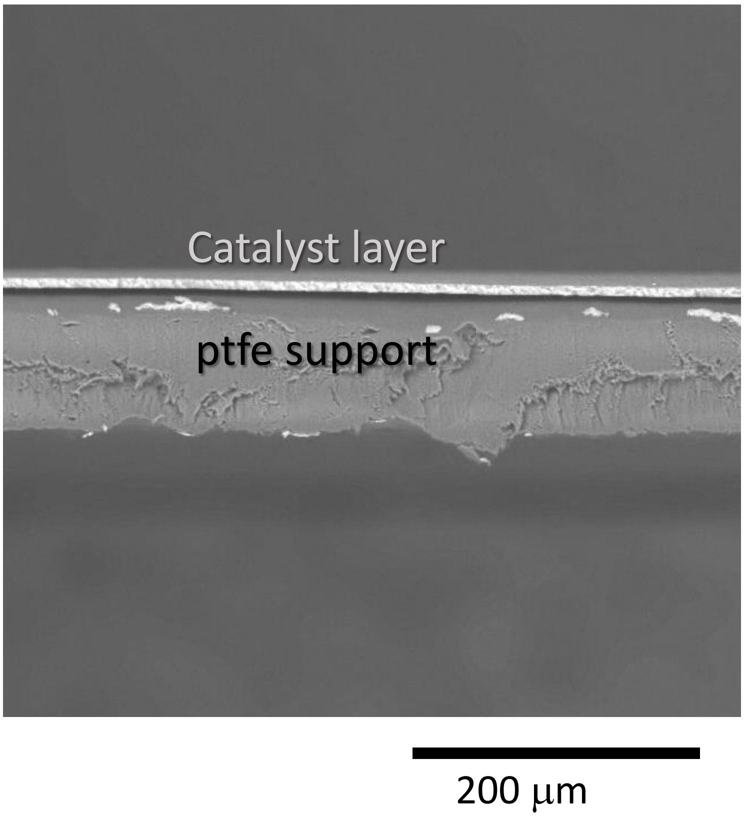


***Figure S4:*** *SEM of model catalyst layer deposited on top of a ptfe film. Catalyst loading 200* $\mu g_{Pt}$ $cm^{-2}$.

## S5. Derivation of electrical collection in the presence of voids

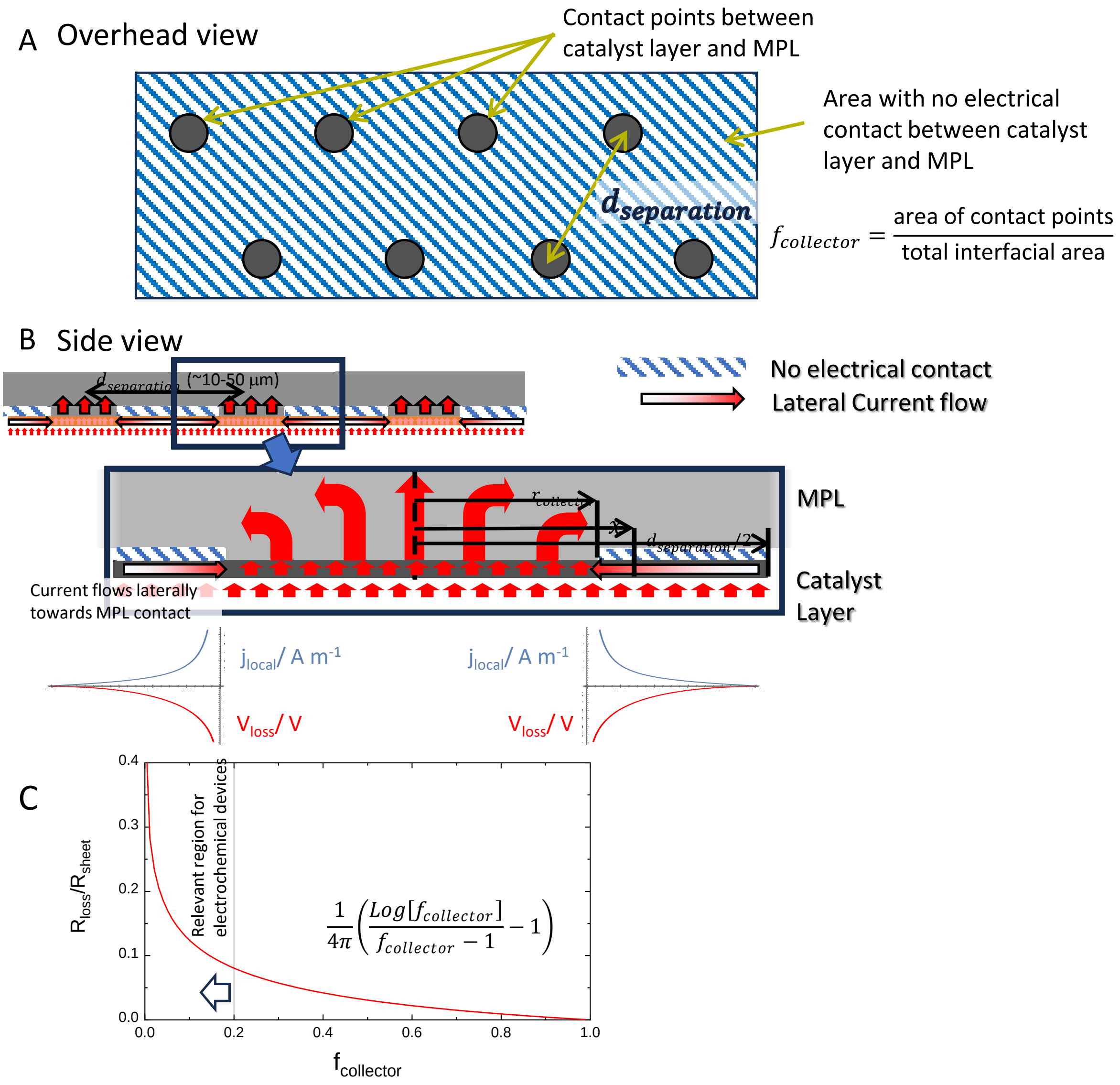


*Figure S5: A) Overhead view showing geometry of contact points between catalyst layer and MPL; B) Cartoon of cross section of MPL/catalyst layer in which voids or poor contact between the MPL and catalyst layer force lateral current flow through the catalyst layer to a point at which the MPL makes contact with the catalyst layer; C) Dimensionless plot of the effective contact resistance scaled by the sheet resistance as a function of the area of the contact region scaled by the area of the current collection region.*

The model for calculating the effective area specific resistance associated with lateral current flow within the catalyst layer assumes a catalyst layer which is much thinner than the lateral extent of current flow such that it can be treated as a film which is characterized by a sheet resistance, $R_{sheet}$ ($\Omega$ square$^{-1}$). A cartoon of the configuration is given in Figure S5A&B. For simplicity, only the catalyst layer and points at which the MPL makes contact with the catalyst layer (current collector) are shown. Figure S5A shows an overhead view in which the points at which current flows through contact points between catalyst layer and MPL are shown as filled circles In between the contact point there are

areas through which no electrical current can pass to the MPL (striped bars), associated with gas transport voids or electrically insulating layers (e.g. ionomer, ptfe etc) between the catalyst layer and the MPL. Figure S5B shows a cross section of the layer which transects through the contact points and areas through which no current flow. In these latter regions the current generated in the catalyst layer (vertical upward arrows below the catalyst layer) flow radially towards the current collector regions and then into the MPL through the contact areas. The MPL makes contact with the catalyst layer at current collector regions with a spacing of $d_{separation}$. Each current collector collects current from a circular region of area $\pi\left({d_{separation}}/{2}\right)^2$. The area of the current collector is a fraction, $f_{collector}$, of the total interfacial area (i.e the current collector has a radius of $r_{collector} = {1}/{2}\sqrt{f_{collector}}d_{separation}$). The current density, $j$ (A m$^{-2}$), flowing into the catalyst layer is assumed to be uniform with position i.e. we make the simplifying assumption that the lateral potential drop in the catalyst layer does not affect the local electrokinetics and the current density flowing into the catalyst layer is constant everywhere. Current flowing into the catalyst layer immediately below the current collector area flows directly into the contact (i.e. does not flow laterally), whereas for those at a distance ($x$) beyond the current collector( $r_{collector} < x < {1}/{2}\, d_{separation}$ ), the current must flow laterally in a radial direction towards the current collector. The lateral current is greatest immediately adjacent to the current collector as the lateral current flow integrates the current from all positions further away from the collector. The linear current density, $j_{local}$ (A m$^{-1}$) at a distance $x$ from the centre of the current collector represents the total current sourced (or sunk) from the area at a distance greater than $x$ and which has to pass through the circular boundary at a distance of $x$ from the centre of the domain:

$$j_{local} = \frac{j\pi\left(\left(d_{separation}/2\right)^2 - x^2\right)}{2\pi x} \qquad \text{Eq S1}$$

The shape of $j_{local}$ is shown below the cartoon in Figure S5B as a function of position away from the collector. The local potential gradient, $V_{Local}$ (V m$^{-1}$) due to the current flowing through the catalyst layer of sheet resistance $R_{sheet}$($\Omega$ square$^{-1}$) at a position $x$ from the center of the domain is simply this local current multiplied by the sheet resistance:

$$V_{local} = -\frac{1}{2} j R_{sheet}\left(\frac{\left(d_{separation}/2\right)^2}{x} - x\right) \qquad \text{Eq S2}$$

The total voltage loss, $V_{loss}$(V) seen by the current flowing laterally from the outermost area to the edge of the current collector is then the integral of this function from the furthest point to the edge of the current collector:

$$V_{loss} = \int_{d_{separation}/2}^{r_{collector}} -\frac{1}{2} j R_{sheet} \left( \frac{\left(\frac{d_{separation}}{2}\right)^2}{x} - x \right) dx = -\frac{1}{16} j R_{sheet} \left( d_{separation}{}^2 \left( 1 - \frac{4 r_{collector}{}^2}{d_{separation}{}^2} + \text{Log}\left[ \frac{4 r_{collector}{}^2}{d_{separation}{}^2} \right] \right) \right) \quad \text{Eq S3}$$

This voltage loss is also shown as a graph below the cartoon in Figure S5B. The effective resistance, $R_{loss}$ ($\Omega$) of the lateral current flow out of **one** current collector is then this voltage loss divided by the total current collected from the region surrounding that current collector. Rewriting $r_{collector}$ in terms of the fraction of available area ($f_{collector}$) we obtain:

$$R_{loss} = \frac{V_{loss}}{\pi\left(\left(d_{separation}/2\right)^2 - r_{collector}{}^2\right) j} = \frac{R_{sheet}}{4\pi} \left( \frac{Log[f_{collector}]}{f_{collector} - 1} - 1 \right) \quad \text{Eq S4}$$

This result shows that the resistive loss associated with lateral current flow to one current collection area is only dependent on the *relative area* of the contact and not the size of the collection area. Figure S5C shows the dimensionless plot of $\frac{R_{loss}}{R_{sheet}}$ as a function of $f_{collector}$. As an example, if we assume that only 10% of the area is in contact with MPL, we see that resistance associated with each contact point is about 12% that of the sheet resistance.

The number of current collector points per unit area is inversely proportional to the square of the separation between the contact points and so the total loss, $R_{extra}$ is decreased by the number of contact points per unit area:

$$R_{extra} = \frac{Resistance\ per\ contact}{number\ of\ contacts} = \frac{\frac{R_{sheet}}{4\pi}\left(\frac{Log[f_{collector}]}{f_{collector} - 1} - 1\right)}{\left(1/\left(\pi\left(d_{separation}/2\right)^2\right)\right)} = \frac{1}{16} d_{separation}{}^2 R_{sheet} \left( \frac{Log[f_{collector}]}{f_{collector} - 1} - 1 \right) \quad \text{Eq S5}$$

Which is the result presented in equation 1 in the main paper.

## S6. Estimation of total reaction orders for ORR on Pt, PtNi, PtZn and PtCo

In Figures S6-S9 we show the individual plots of catalyst performance (specific current density) corrected for hydrogen crossover for each of the catalyst tested as a function of oxygen partial pressure (corrected for water vapour). Hydrogen crossover correction was performed by adding the hydrogen crossover current to the current density and assumes all crossover hydrogen is oxidised at the cathode. The results are shown for two independent MEAs.

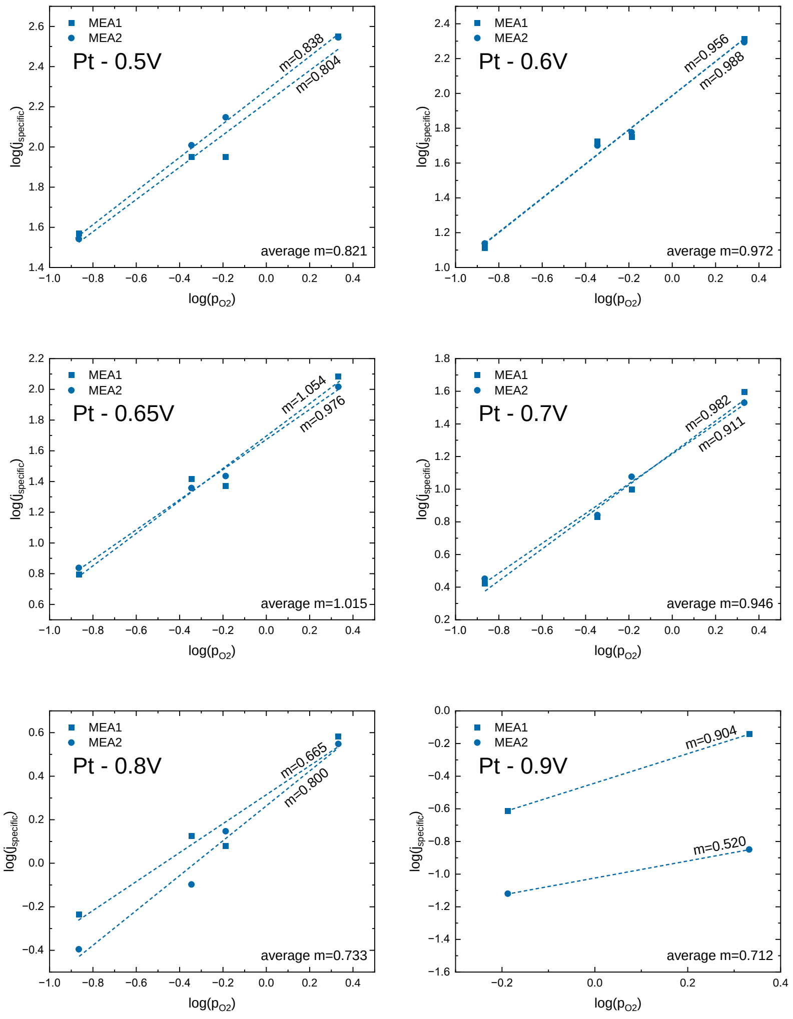


***Figure S6***: *Total reaction order for Pt/C electrocatalyst in fuel cell composed of 5.2 - 7.1 $\mu g_{Pt}cm^{-2}$ on anode and cathode. Results from two MEAs shown. The polarization curves were corrected for water partial pressure, oxygen equilibrium potential and $H_2$ crossover before determining the reaction order. 80/80/73 °C (cell temperature/anode bubbler/cathode bubbler). Oxygen pressure normalized to $p^{\Theta}$=1 bar.*

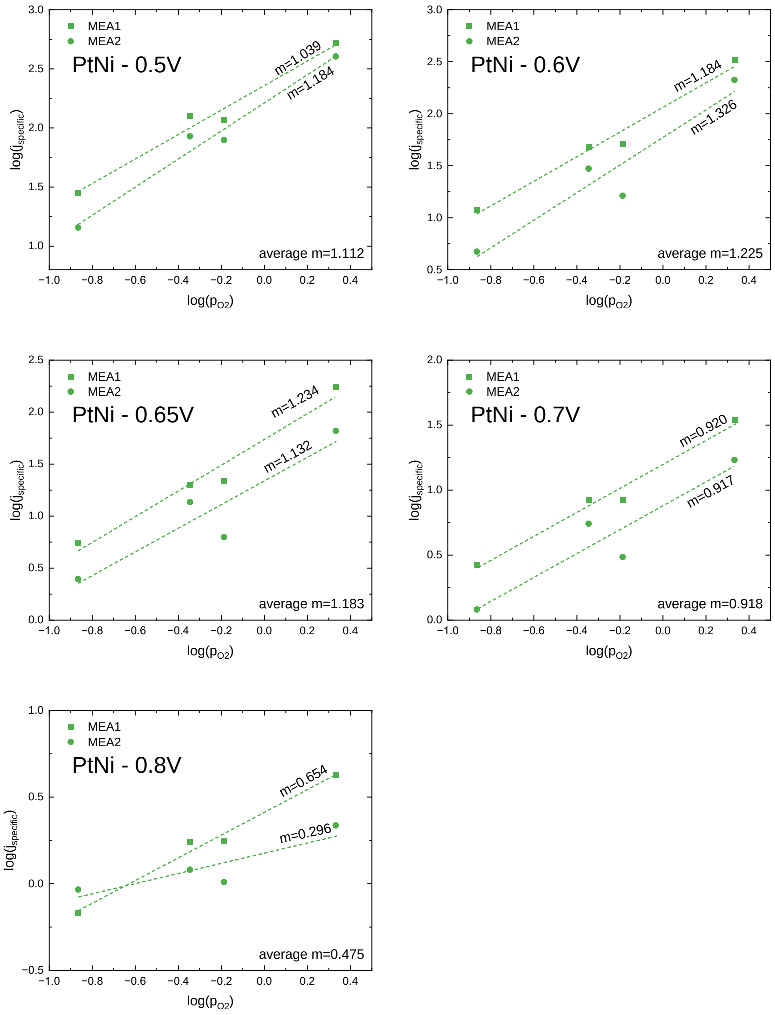


***Figure S7****: Total reaction order for PtNi/C electrocatalyst in fuel cell composed of 5.2 – 7.1 $\mu g_{Pt}cm^{-2}$ on anode and cathode. Results from two MEAs shown. The polarization curves were corrected for water partial pressure, oxygen equilibrium potential and $H_2$ crossover before determining the reaction order. 80/80/73 °C (cell temperature/anode bubbler/cathode bubbler). Oxygen pressure normalized to $p^{\Theta}$=1 bar.*

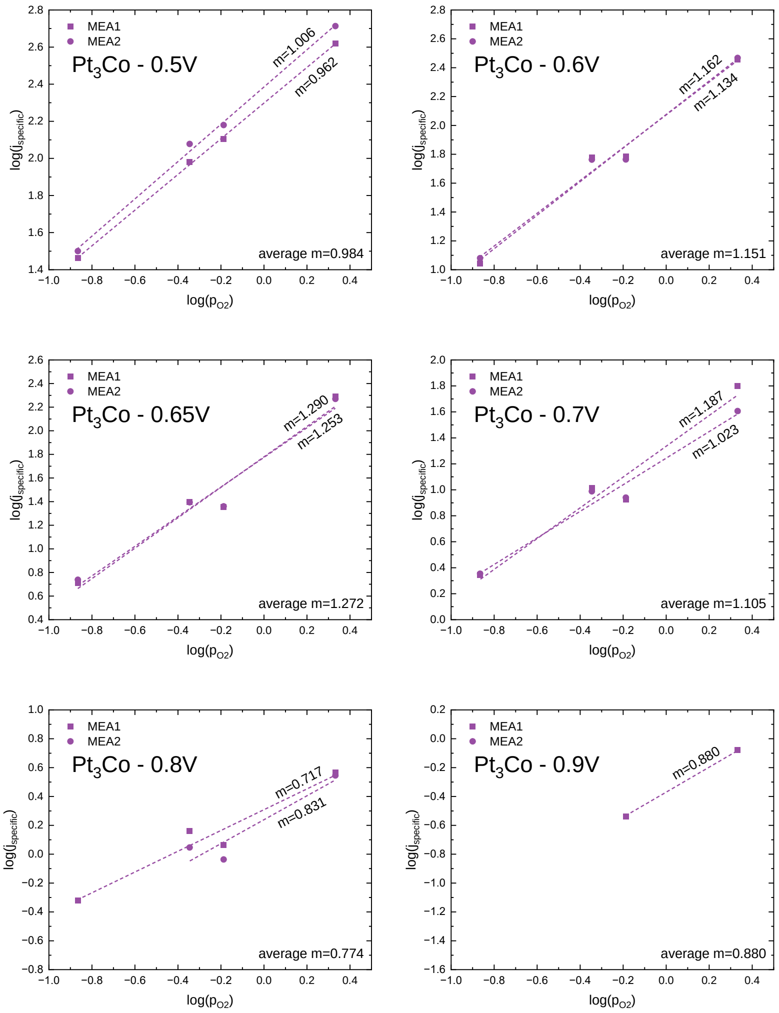


***Figure S8****: Total reaction order for $Pt_3Co$/C electrocatalyst in fuel cell composed of 5.2 – 7.1 $\mu g_{Pt}cm^{-2}$ on anode and cathode. Results from two MEAs shown. The polarization curves were corrected for water partial pressure, oxygen equilibrium potential and $H_2$ crossover before determining the reaction order. 80/80/73 °C (cell temperature/anode bubbler/cathode bubbler). Oxygen pressure normalized to $p^{\Theta}$=1 bar.*

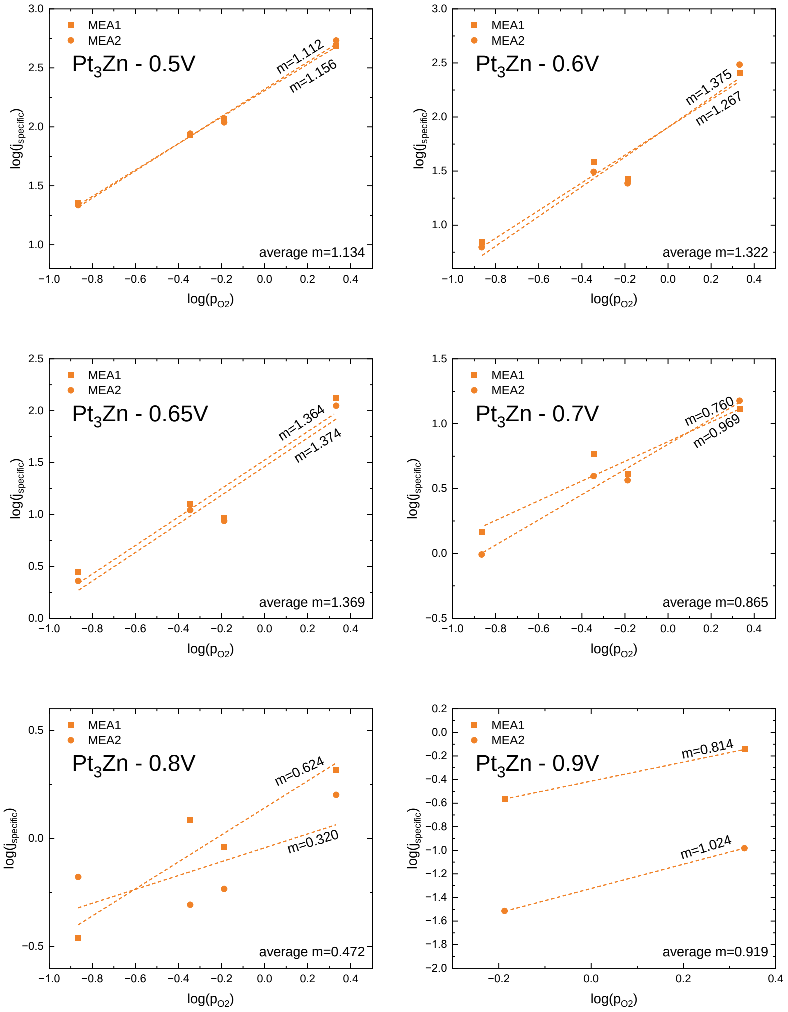


***Figure S9**: Total reaction order for $Pt_3Zn$/C electrocatalyst in fuel cell composed of 5.2 – 7.1 $\mu g_{Pt}cm^{-2}$ on anode and cathode. Results from two MEAs shown. The polarization curves were corrected for water partial pressure, oxygen equilibrium potential and $H_2$ crossover before determining the reaction order. 80/80/73 °C (cell temperature/anode bubbler/cathode bubbler). Oxygen pressure normalized to $p^{\ominus}$=1 bar.*

## S7. Comparison of different approaches for low loading electrodes.

A comparison of the different approaches used to produce low loading electrodes and whether they provide an idea of reproducibility (error bars on performance) and directly assess platinum loading, indirectly assess platinum  loading or use of "dead reckoning" to assess the loading is provided in the Table below.

| Group | Method | Error Bars on performance | Direct loading measurement | Indirect loading estimate | Comments |
|---|---|---|---|---|---|
| This study | Filtration with inclusion of nanoporous layer | Yes | XRF | | |
| Cavarroc et al.[6] | Magnetron Sputtering | No | RBS | | Rutherford Backscattering Spectroscopy |
| Tian et al.[8] | DC and RF sputtering on VACNTs | No | | Weighted using microbalance | Vertically aligned carbon nanotubes |
| Liu et al.[9] | Electrospinning | | | Varying Electrospinning time and measuring layer thickness | Error bars for nanotube wall thickness + diameter |
| Shukla et al.[18] | Inkjet Printing | Yes | XRF | Measured using gravimetric analysis | Loading was varied by changing the number of printed passes |
| Cogneli et al.[21] | Sputter Deposition (RF) | No | | Used profilometry measurements on reference Si wafer | Controlled by sputtering time |
| Martin t al.[22] | Electrospraying | No | | | Not mentioned in previous paper either |
| Wang t al.[23] | Electrospinning/ Electrospraying (E/E) system | No | TGA | | Comparing weight before and after exposure to 900°C |
| Gruber et al.[24] | Sputter Deposition | No | | Referenced to Pt deposited on Si wafer | Controlled by sputtering time and material density |
| Saha et al (2006).[25] | Dual Ion Beam Assisted Deposition | No | | Measured Pt layer thickness | |
| Brodt et al.[26] | Electrospinning | No | | Calculated using total weight of nanofiber and the weight-fraction of catalyst used in ink | Controlled by varying duration of electrospinning |
| Saha et al (2011).[27] | Piezo-electric Printing | No | Neutron Activation Analysis | | |
| Millington et al.[28] | Ultrasonic Spraying | No | | Varying the number of passes | The study they reference for the method used ICP to confirm loading, nothing mentioned in this paper |
| Dang et al.[29] | Facile Pulse Electrodeposition | No | Atomic absorption spectroscopy | | |
| Xiong et al.[30] | Modified thin film method | No | | Weighing before and after applying ink and drying | |
| Yu et al.[31] | Reactive Spray Deposition | No | | | |
| Brault et al.[32] | Plasma Sputtering Deposition | No | RBS | | |

## S8. Local reaction conditions in the catalyst layer.

Under operating conditions, when the cathode Is producing water, we would expect the vapour in the cathode catalyst layer to become saturated with water vapour. This has an effect on the effective oxygen partial pressure and decreases the available partial pressure by different amounts depending on whether air of pure oxygen is used. Table S2 shows the expected oxygen partial pressure based on the relative humidity at a temperature of 80°C. Note that at 100% RH the difference in oxygen partial pressure between the 1 bar $O_2$ and 2.5 bar air cases almost halves from 0.194 bar to 0.101 bar. This effect is also seen in Figure S10A and B which show log-log plots of the performance results from Table 1 (main paper) at 0.65 V versus oxygen partial pressure under the assumption of either 75% or 100% relative humidity. Interestingly, if local relative humidity were supersaturated to 127% then the oxygen partial pressures would be the same in both conditions, suggesting that supersaturation of water vapour in the catalyst layer may be an important issue in these layers. This effect could also be achieved by raising the catalyst layer temperature to 86°C at which point the water partial pressure (100%RH) would be the same as 127% of the value at 80°C. Hence the similarity of the results under these two conditions could be explained by either (a) supersaturation of water vapour within the catalyst layer; or (b) increase of local temperature in the catalyst layer with saturation to 100%RH at that temperature; or a combination of both.

***Table S2: Oxygen partial pressure (bar) as a function of relative humidity and initial gas composition.***

| | 75%RH | 100%RH | 127%RH |
|---|---|---|---|
| 1 atm(abs) air | 0.135 | 0.110 | 0.084 |
| 2.5 atm(abs) air | 0.450 | 0.425 | 0.399 |
| 1 atm(abs) $O_2$ | 0.644 | 0.526 | 0.399 |
| 2.5 atm(abs) $O_2$ | 2.144 | 2.026 | 1.899 |

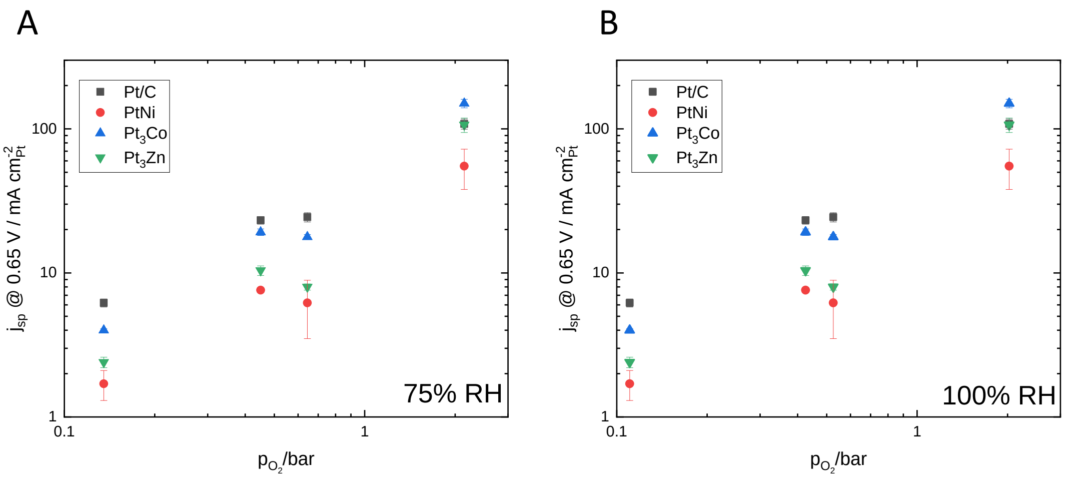


***Figure S10**: Log-Log plots of $j_{sp}$ (Table 1, main paper) versus oxygen partial pressure under the assumption of either (A) 75% or (B) 100% RH in the catalyst layer.*
